# Effect of posture on anorectal manometric measurements in female patients with fecal incontinence and rectoanal intussusception

**DOI:** 10.1186/s12876-022-02581-7

**Published:** 2022-11-22

**Authors:** Akira Tsunoda, Tomoko Takahashi, Ikuko Osawa

**Affiliations:** 1 Department of Gastroenterological Surgery, Kameda Medical Center, 929 Higashi-Cho, Kamogawa City, Chiba 296-8602 Japan; 2 Department of Clinical Laboratory, Kameda Medical Center, 929 Higashi-Cho, Kamogawa City, Chiba 296-8602 Japan

**Keywords:** Fecal incontinence, Rectoanal intussusception, Squeeze pressure

## Abstract

**Purpose:**

This study aimed to investigate the influence of erect position on anorectal manometry in patients with rectoanal intussusception (RAI).

**Methods:**

This was a single center prospective observational study. Eighty female patients with fecal incontinence (FI) who underwent defecography between 1st January 2016 and 30th April 2022 were included. The effect of posture on commonly measured parameters during manometry was assessed in the left-lateral and erect positions. The severity of FI was assessed using FI Severity Index (FISI).

**Results:**

Defecography showed that 30 patients had circumferential RAI (CRAI), and 50 had non-CRAI. There were no significant differences in age, parity, FI type, and FISI scores between the groups. However, FISI scores were significantly lower in 51 patients with passive FI than 12 patients with mixed FI type [21 (8–38) vs. 32 (8–43), *P* = 0.007]. Endo-anal ultrasound showed no significant difference in the incidence of sphincter defects between the groups. Maximum squeeze pressure was significantly lower in the erect position than in the left-lateral position in the CRAI patients [119 cm H_2_O (59‒454 cm H_2_O) vs. 145 cm H_2_O (65‒604 cm H_2_O), *P* = 0.006] however, this finding was not observed in the non-CRAI group and the subgroup of anterior RAI patients. In either group, maximum resting pressure, defecation desire volume, and maximum tolerated volume were significantly higher, while anal canal length was significantly shorter in the erect position than in the left-lateral position, respectively.

**Conclusion:**

Voluntary contraction in female FI patients with CRAI was suppressed in the erect position.

**Supplementary Information:**

The online version contains supplementary material available at 10.1186/s12876-022-02581-7.

## Introduction

Rectal intussusception is an infolding of the rectal wall that may occur during defecation. In patients with anorectal dysfunction, rectal intussusception is a common finding in defecography [1]. Rectal intussusception is frequently observed in older female patients and may cause symptoms of obstructed defecation and fecal incontinence (FI) [2, 3]. Rectal intussusception can be classified into rectorectal intussusception and rectoanal intussusception (RAI). RAI is diagnosed when the apex of the rectal intussusception is impinging on the internal anal orifice or intra-anal [4]. Routine defecography is usually performed on patients under examination for FI in tertiary specialist centers [5, 6].

Anorectal manometry is commonly used to investigate FI. Conventionally, measurements are performed with the patient in the left-lateral position [7] however, we observed that patients with RAI usually report FI episodes in the erect position rather than the lateral position. Thekkinkattil et al. [8] reported that incontinence severity was better correlated with resting pressure in the erect position than in the left-lateral position in FI patients with idiopathic and non-idiopathic pathogenesis who did not undergo defecography. Also, maximum resting pressure (MRP) was reported to be higher in the erect position than in the lateral position in healthy subjects however, maximum squeeze pressure (MSP) was not different between the two postures [9].

To date, the influence of erect position on anorectal manometry in FI patients with RAI and/or rectocele remains unclear. This study aimed to compare anorectal physiologic measurements for both the erect and left-lateral positions.

## Methods

This was a prospective study conducted in a single institution. Between 1st January 2016 and 30th April 2022, defecography was performed on 292 females who presented with FI. The findings included: external rectal prolapse (n = 127), rectal intussusception (RI) alone (n = 38), RI with rectocele (n = 28), RI with enterocele (n = 12), RI with rectocele and enterocele (n = 13), rectocele alone (n = 30), rectocele with enterocele (n = 7), mucosal prolapse (n = 20), and others (n = 17). A total of 128 patients with RI and/or rectocele met a condition of the study. Of these, 46 patients were not asked to participate in the study due to forgetting of researchers, two refused and consequently 80 patients were included in the study (Additional file 1), and written informed consent was obtained from all subjects. The remaining 164 patients with other abnormalities were excluded from the study. This study was approved by the hospital’s Ethical Committee (approval number: 16-102-200605). During a period of 6 years, the same standard protocol was followed. FI severity was assessed using FI Severity Index (FISI) [10]. FI has three main subtypes: passive, urge, and a combination of both symptoms. Passive FI is defined as involuntary loss of liquid or solid stool without awareness. Urge FI is defined as the involuntary loss of solid or liquid stool despite attempts to prevent defecation, often with strong sense of urgency [11]. Constipation severity was evaluated using Constipation Scoring System (CSS) [12]. Some parts of the data in this study were reported in the non-English literature [13].

### Defecography

A standardized defecography technique was used. Proctograms were evaluated using the criteria proposed by Shorvon et al. [4]. Briefly, RAI was diagnosed when the RI apex was impinging on the internal anal orifice (level I) or was intra-anal (level II), based on images taken during maximal straining defecation. As opposed to RAI, rectorectal intussusception was diagnosed if the apex remained intrarectal and was not impinging on the internal anal orifice. A rectocele ≥ 2 cm in diameter was considered abnormal. The size was calculated in standard fashion in the anterior­posterior dimension by measuring the distance between the actual most ventral part of the anterior rectal wall and an extrapolated line indicating the expected position of the rectal wall [14]. Enterocoele was diagnosed when the extension of the bowel loop was located between the vagina and the rectum. Images from defecography were analyzed by one of the authors (T. T.), who is experienced in the evaluation and was blinded at that time to the symptomatology of the individual patients.

### Physiological measurements

**Fig. 1:**
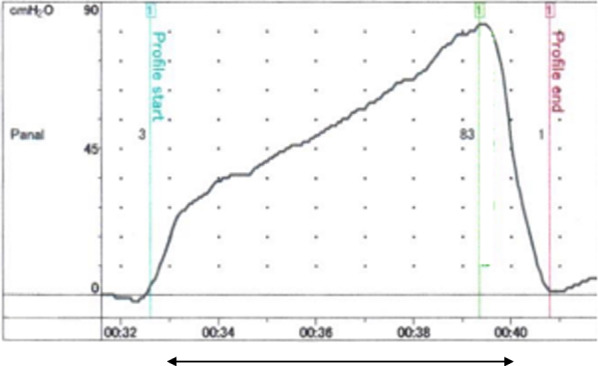
Anal canal pressure profile. Anal canal length was measured based on the appearance of high pressure zone (arrow).

Each patient underwent laboratory evaluations of the anal sphincter function. Air-Charged catheters (Urodynamic System Solar, Edaptechnomed Co. Ltd, Tokyo) was used to measure the MRP and MSP [15]. The position of the transducer in the rectum was confirmed by recording pressures of 10 mm H_2_O or less. The transducer was left in place for 2‒3 min to allow the anal canal to accommodate to its presence. The anal canal length and MRP were determined by pulling the transducer slowly through the anal canal at about 5 mm per 3 s, not to activate the anal-external sphincter continence reflex [16]. The probe recorded MRP three times, and the median value was adopted. The anal canal length was determined using internal definition agreed by three authors as the difference between the measurements at the beginning of the resting pressure rise and the end of the pressure fall (Fig. 1). The probe was then re-introduced, and the patients were asked to squeeze their anal muscles maximally each time the transducer was pulled through the anal canal in 0.5 cm increments, and the maximum value was adopted. In-house normal values of MRP and MSP in healthy volunteers are 60‒120 cm H_2_O and 150‒300 cm H_2_O, respectively when measured in the left-lateral position (no reference).

The sensation of the rectum was measured by placing a balloon, with its lower extremity 5 cm from the anal verge. Rectal perception of distention was examined by slowly inflating the balloon to a maximum volume of 300 ml, and the defecation desire volume (DDV) and maximum tolerated volume (MTV) were measured. Physiological measurements were performed by a single female clinical technologist (I.O.), initially in the left-lateral position and then in the erect position. The outcome was interpretated by other researchers.

### Endo-anal ultrasound (EAUS)

EAUS was performed using a B-K Medical ultrasound system, an endoprobe with a 6–16 MHZ radial transducer (Type 2052) in the left-lateral position (B-K Medical, Herlev, Denmark).

### Statistical analysis

Data for continuous variables were presented as median (range) and tested using Mann–Whitney *U* test for unpaired data and Wilcoxon signed-rank test for paired data. The Chi-square test or Kruskal–Wallis test was used for categorical variables. The data were analyzed with SPSS v26 (IBM Corp., Armonk, NY, USA). Statistical significance was considered at *P* < 0.05.

## Results

### Patients’ characteristics

The characteristics of the study participants are shown in Table 1. Thirty patients (37.5%) had circumferential rectoanal intussusception (CRAI), and 50 (62.5%) had non-CRAI. The majority of patients had passive FI in either the group and the incidence was 67% (20/30) in the CRAI group and 62% (31/50) in the non-CRAI group. The frequency of three FI types was not significantly different between the groups. Forty-three percent (13/30) of the CRAI group and 40% (20/50) of the non-CRAI group had previous history of pelvic surgeries. Three patients with irritable bowel syndrome (IBS) with predominant diarrhea diagnosed using Roma III criteria were included in the non-CRAI group. None of the patients had IBS with predominant constipation. There were significant differences in FISI scores among patients with three FI types (*P* = 0.027), and the scores were significantly lower in patients with passive FI than those with mixed FI type [21 (8–38) vs. 32 (8–43), *P* = 0.007]. There were no significant differences in CSS scores among patients with three FI types. FISI scores and CSS scores were not significantly different between the groups, respectively.

**Table 1:** Characteristics of patients

	Total (*n* = 80)	CRAI (*n* = 30)	non-CRAI (*n* = 50)	*P *value^a^
Age, years	75 (39‒92)	76 (49‒92)	73 (39‒92)	0.10*
Parity	2 (0‒5)	2 (0‒4)	2 (0‒5)	0.56*
Type of fecal incontinence				
Passive/urgent/mixed	51/17/12	20/5/5	31/12/7	0.73^†^
Previous pelvic surgery	33	13	20	0.77^†^
Hysterectomy	13	5	8	
Surgery for hemorrhoids	11	5	6	
Surgery for POP	8	3	5	
Surgery for anal fistula	1	0	1	
Irritable bowel syndrome	3	0	3	0.29
FI severity index scores	21 (8‒59)	24 (8‒38)	20 (8‒59)	0.53*
CSS scores	6 (0‒20)	6 (0‒20)	6 (0‒17)	0.85*

Values are presented as *n* or median (range)

*CRAI* circumferential rectoanal intussusception, *POP* pelvic organ prolapse, *FI* fecal incontinence, *CSS* constipation scoring system

^a^CRAI versus non-CRAI group

*Mann–Whitney *U* test

^†^chi-square test

**Fig. 2:**
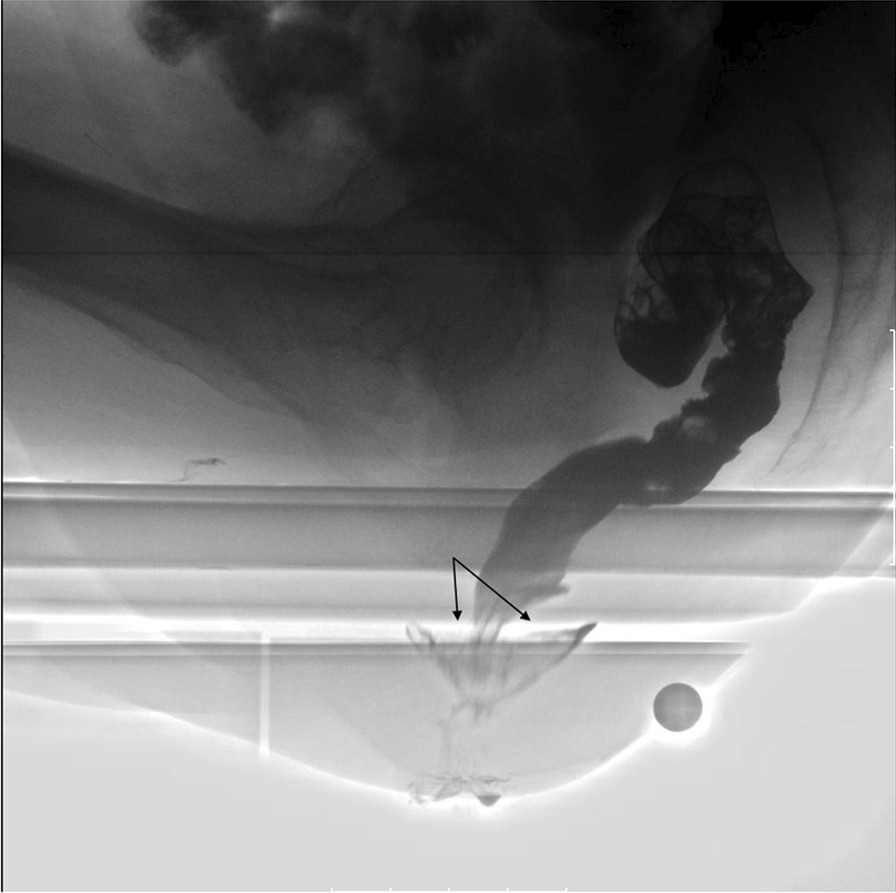
Defecographic finding of circumferential rectoanal intussusception (solid arrow)

**Fig. 3:**
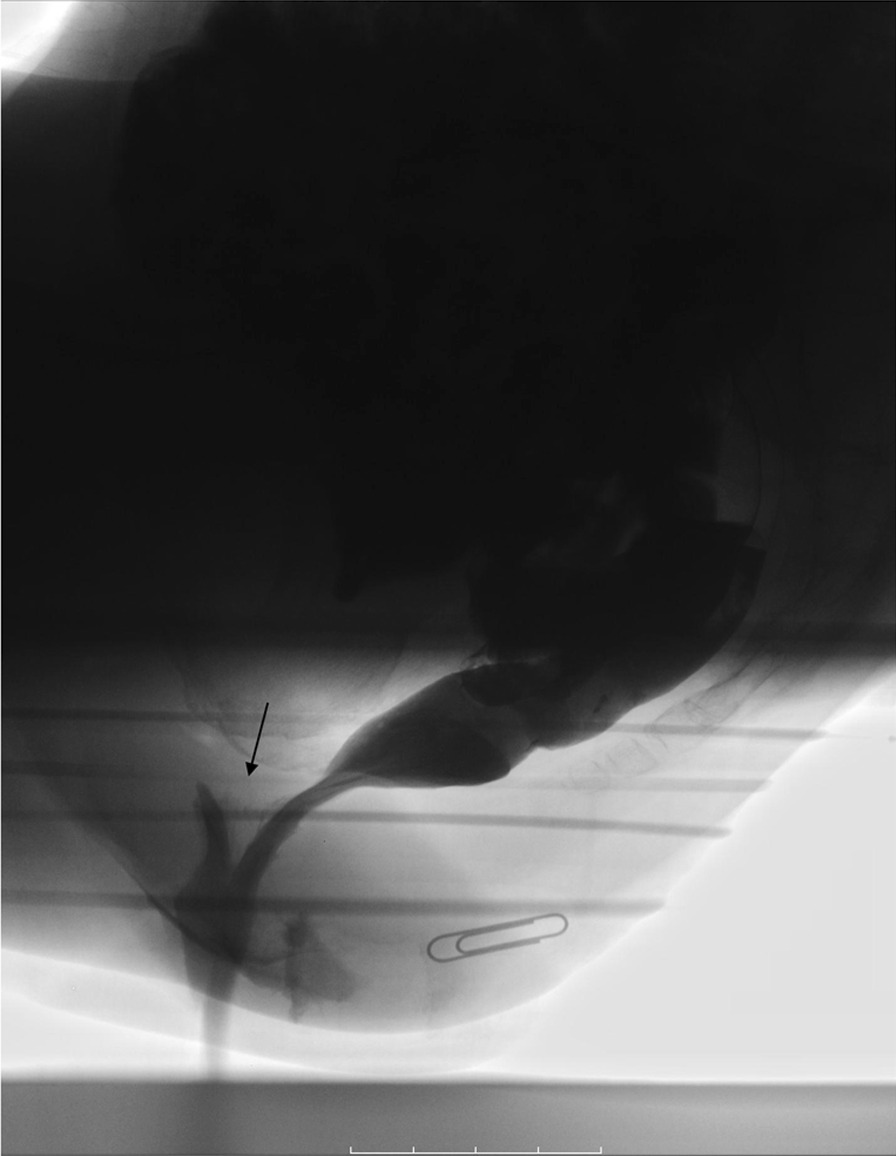
Defecographic finding of anterior rectoanal intussusception (solid arrow)

The image findings and physiological measurements are shown in Table 2. The defecography findings in the CRAI group included CRAI alone (Fig. 2) (n = 22) and CRAI with rectocele (n = 8), and those in the non-CRAI group included anterior RAI alone (Fig. 3) (n = 4), anterior RAI with rectocele (n = 5), rectorectal intussusception alone (n = 6), and rectocele alone (n = 15). The posterior rectal intussusception was not found in either the group. The incidence of level I and level II CRAI was almost equal (14/30 vs. 16/30, respectively). EAUS findings showed that all patients had intact anal sphincters and no significant difference in the incidence of the abnormalities between the CRAI and non-CRAI groups. Among 71 patients who underwent EAUS, 15% (4/27) of the CRAI group and 11% (5/44) of the non-CRAI group had partial internal or external sphincter defects. Partial internal sphincter defect was found on one patient in the CRAI group. There were no significant differences in the physiological measurements between the groups. The median anal canal length in either the group was almost equal (4.6 cm in the CRAI group vs. 4.5 cm in the non-CRAI group). The median MRP was just a little smaller (7 cm H_2_O) in the CRAI group than that in the non-CRAI group. The same was the case with the median MSP (5 cm H_2_O smaller in the CRAI group). The median DDV in the CRAI and non-CRAI group was 85 ml and 80 ml, respectively. Similarly, the median MTV was 170 ml and 200 ml, respectively.

**Table 2:** Image findings and physiological measurements

	CRAI (*n* = 30)	non-CRAI (*n* = 50)	*P *value^a^
Defecographic findings			
CRAI (level I/II)	22 (9/13)	–	< 0.0001^†^
CRAI (level I/II) + rectocele	8 (5/3)	–	
Anterior rectoanal intussusception	–	4	
Anterior rectoanal intussusception + rectocele	–	5	
Rectorectal intussusception	–	6	
Rectocele alone	–	15	
Endoanal ultrasound			
No sphincter defect	23	39	0.63^†^
Partial defect of internal anal sphincter	1	0	
Partial defect of external anal sphincter	3	5	
Not examined	3	6	
Physiological measurements (left-lateral position)			
Anal canal length, cm	4.6 (3.2‒6.6)	4.5 (2.5‒6.5)	0.34*
Maximum resting pressure, cm H_2_O	51.5 (34.9‒124.8)	58.5 (19.0‒114.4)	0.98*
Maximum squeeze pressure, cm H_2_O	145.3 (65.1‒604.0)	150.5 (45.7‒366.7)	0.53*
Defecation desire volume, ml	85 (50‒250)	80 (20‒280)	0.32*
Maximum tolerated volume, ml	170 (80‒300)	200 (70‒3009	0.71*

Values are presented as *n* or median (range), unless otherwise indicated

*CRAI*, circumferential rectoanal intussusception; level I, descent onto the anal sphincter/anal canal; level II, descent into the anal sphincter/anal canal

^a^CRAI versus non-CRAI group

*Mann–Whitney *U* test

^†^Chi-square test

### Comparison of anorectal physiology in different position

#### The effects of posture on physiological measurements in the 80 FI patients (Table 3)

**Table 3:** Physiological measurements in female patients with fecal incontinence in the left-lateral and erect positions (*n* = 80)

	Left-lateral position	Erect position	*P* value*
Anal canal length, cm	4.5 (2.5‒6.6)	4.2 (1.8‒6.8)	< 0.0001
Maximum resting pressure, cm H_2_O	54.8 (19.0‒124.8)	61.5 (18.7‒156.0)	< 0.0001
Maximum squeeze pressure, cm H_2_O	149.9 (45.7‒604.0)	148.0 (44.0‒454.4)	0.07
Defecation desire volume, ml	80 (20‒280)	125 (30‒300)	< 0.0001
Maximum tolerated volume, ml	188 (70‒300)	215 (30‒300)	< 0.0001

Values are presented as median (range)

*Wilcoxon signed rank test

The MRP, DDV, and MTV were significantly higher in the erect position than in the left-lateral position, respectively, while anal canal length was significantly shorter in the erect position than in the left-lateral position. The MSP in the left-lateral position was not significantly different from that in the erect position in all 80 FI patients.

#### The effects of posture on physiological measurements in the CRAI and non-CRAI groups (Table 4)

**Table 4:** Physiological measurements in CRAI and non-CRAI groups in the left-lateral and erect positions

	Left-lateral position	Erect position	*P* value*
CRAI (*n* = 30)			
Anal canal length, cm	4.6 (3.2‒6.6)	4.3 (2.2‒6.0)	0.031
Maximum resting pressure, cm H_2_O	51.5 (34.9‒124.8)	58.9 (18.7‒151.1)	0.023
Maximum squeeze pressure, cm H_2_O	145.3 (65.1‒604.0)	118.7 (59.0‒454.4)	0.006
Defecation desire volume, ml	85 (50‒250)	138 (40‒300)	< 0.0001
Maximum tolerated volume, ml	170 (80‒300)	218 (60‒300)	< 0.0001
Non-CRAI (*n* = 50)			
Anal canal length, cm	4.5 (2.5‒6.5)	4.0 (1.8‒6.8)	0.005
Maximum resting pressure, cm H_2_O	58.5 (19.0‒114.4)	65.2 (28.9‒156.0)	0.001
Maximum squeeze pressure, cm H_2_O	150.5 (45.7‒366.7)	164.5 (44.0‒388.7)	0.788
Defecation desire volume, ml	80 (20‒280)	120 (30‒280)	< 0.0001
Maximum tolerated volume, ml	200 (70‒300)	215 (30‒300)	0.007

Values are presented as median (range)

*CRAI* circumferential rectoanal intussusception

*Wilcoxon signed rank test

MRP, DDV, and MTV was significantly higher in either group, and anal canal length was significantly shorter in the erect position than in the left-lateral position. In contrast, MSP was significantly lower in the erect position than in the left-lateral position [119 cm H_2_O (59‒454 cm H_2_O) vs. 145 cm H_2_O (65‒604 cm H_2_O), *P* = 0.006] in the CRAI group. However, this was not observed in the non-CRAI group. When the effect of posture on MSP was evaluated by the type of FI in the CRAI group, MSP was significantly lower in the erect position than the lateral position in patients with either passive (n = 20) or mixed (n = 5) FI however, this was not observed in those with urge FI (n = 5) [91 cm H_2_O (65‒369 cm H_2_O) in the lateral position vs. 124 cm H_2_O (62‒293 cm H_2_O) in the erect position, *P* = 0.65].

**Fig. 4:**
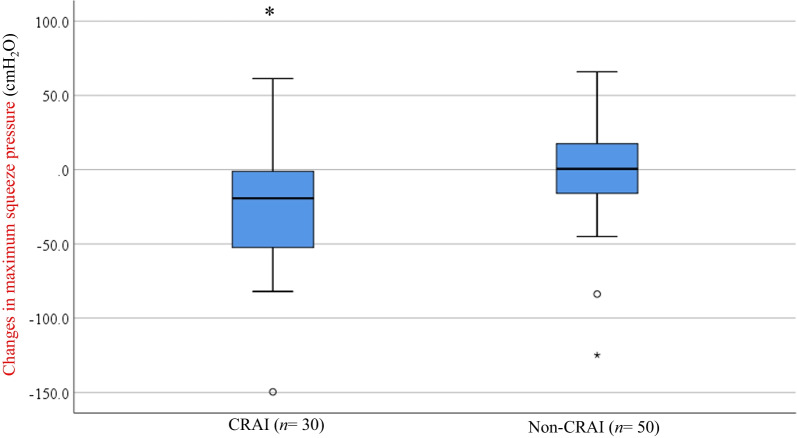
Postural changes in maximum squeeze pressure, which were obtained by subtracting the measurements in the erect position from those of the left-lateral position. *CRAI* circumferential rectoanal intussusception; *P = 0.006 versus non-CRAI group (Mann–Whitney *U* test).

Figure 4 shows the postural changes in MSP; they were obtained by subtracting the measurements in the erect position from those of the left-lateral position in the CRAI and non-CRAI groups. The changes in MSP were significantly different between both groups [− 19.4 cm H_2_O (− 149.6 to 61.4 cm H_2_O) vs. 0.5 cm H_2_O (− 125.0 to 66 cm H_2_O), *P* = 0.006] however, there were no significant differences in the remaining four physiological measurements between them.

**Fig. 5:**
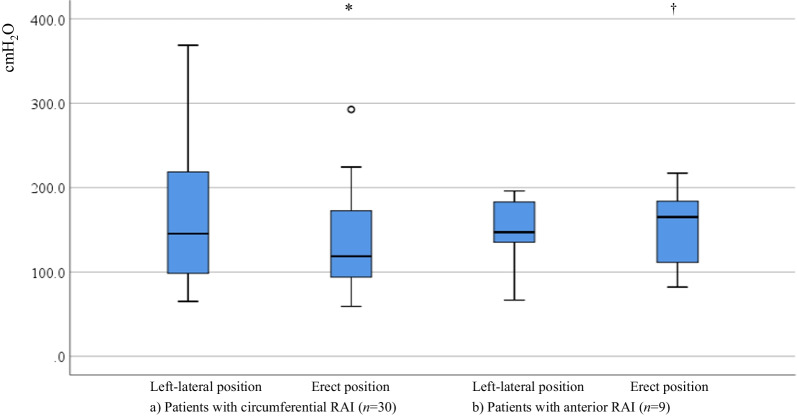
Maximum squeeze pressure in circumferential and anterior RAI groups in the left-lateral and erect positions. *RAI* rectoanal intussusception. *P = 0.006, ^†^*P* = 0.37, versus left-lateral position.

Figure 5 shows the effects of posture on MSP in the CRAI and anterior RAI groups. MSP in the left-lateral position was not significantly different from that in the erect position in patients with anterior RAI (*P* = 0.37).

## Discussion

This study demonstrated that squeeze pressure in female patients with FI and CRAI was significantly lower in the erect position than in the left-lateral position, although this finding was not true of those with non-CRAI. Resting pressure, DDV, and MTV were significantly higher, and anal canal length was significantly shorter in the erect position than in the left-lateral position in either the CRAI or non-CRAI patients.

**Fig. 6:**
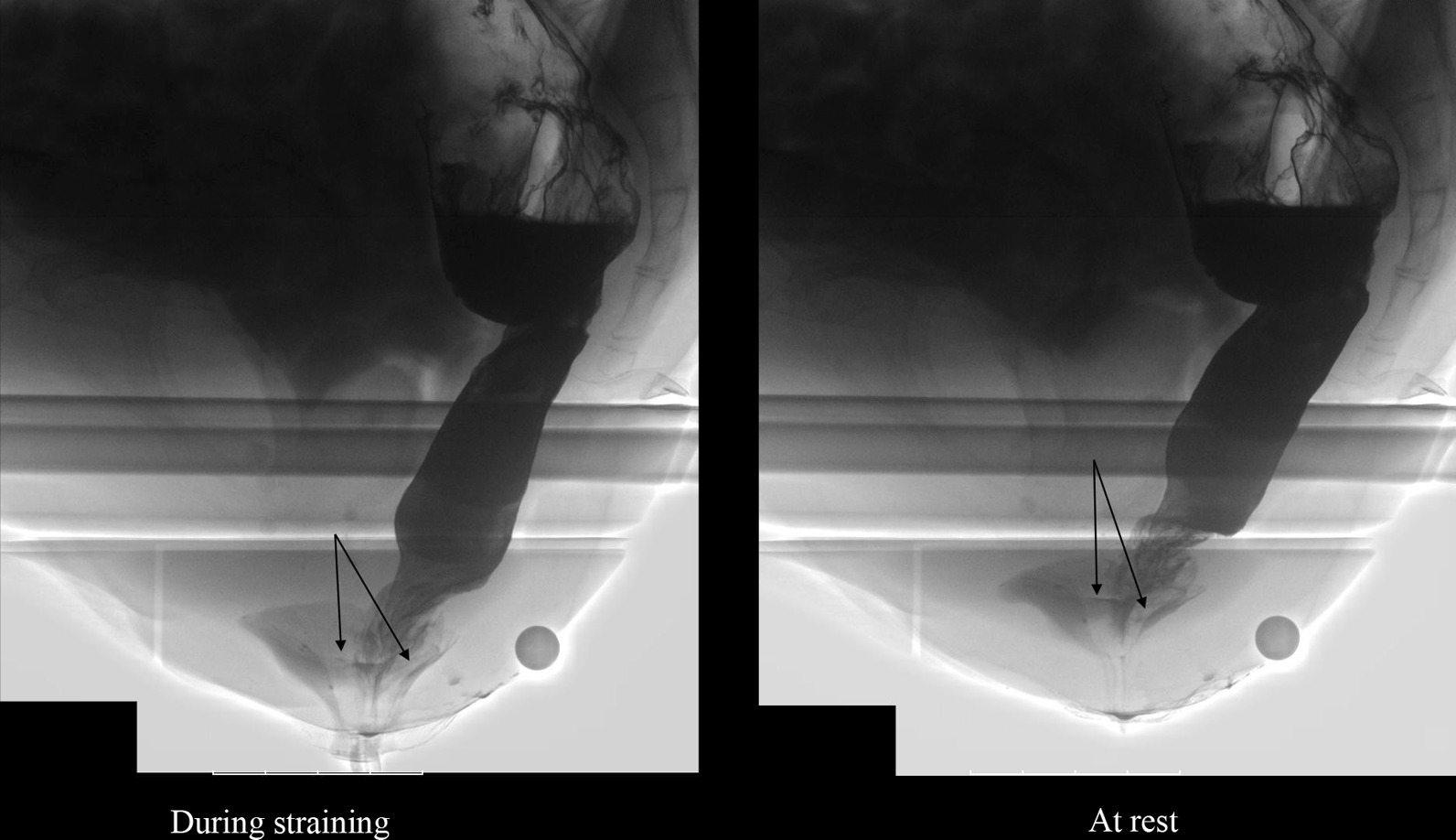
Defecographic findings of circumferential rectoanal intussusception during straining effort and at rest (solid arrow)

Previous studies on the effect of posture on physiological measurements are limited. Johonson et al. [9] reported that posture did not affect squeeze pressure in the study of 27 healthy individuals. This finding was true for all 80 patients with FI in this study however, upon separate examination of the CRAI and non-CRAI groups, squeeze pressure was lower in the erect position than in the lateral position in the CRAI patients. The reason for this finding is unclear however, squeezing the anal canal may be physically inhibited by the intra-anal apex of the “circumferential” RAI but may not be suppressed by “anterior” RAI alone entered during the erect position. Also, it is possible that the higher intra-abdominal pressure created during daily life may expose the lower rectal wall to forces consistent with CRAI development. Notably, CRAI occasionally continued to be observed at rest immediately after CRAI had been observed during straining effort on defecography (Fig. 6). This finding may support the possible existence of the intra-anal apex of RAI at the erect position.

FI was the most common presenting symptom in patients with RAI, in line with previous reports [1, 3, 17]. The cause of FI in patients with RAI has been attributed to reduced resting anal pressure [18, 19], reduced rectal sensation [20], and the inappropriate activation of the rectoanal inhibitory reflex caused by RAI [21]. In this study, two-thirds of patients in the CRAI group had passive incontinence suggesting a dysfunction of the internal anal sphincter, and, notably, only one patient had a partial defect of the internal anal sphincter. Our CRAI patients had fewer incidences of urge and/or mixed incontinence, suggesting dysfunction of the external anal sphincter or a reduction in rectal wall compliance due to chronic irritation of the prolapsed rectal wall [3]. Although squeeze pressure was not lower in the erect position than the lateral position in our CRAI patients with urge FI, their median squeeze pressure measured in the left-lateral position (91 cm H_2_O) was lower than the in-house normal value (150‒300 cm H_2_O). The urge FI may not be caused by the suppression of voluntary contraction in the erect posture, but the underlying reduced function of the external anal sphincter. Also, although squeeze pressure being lower in the erect posture than in the lateral posture in our CRAI patients with passive FI, the passive FI may not be caused by the inhibition of the voluntary contraction theoretically. Nonetheless, squeeze pressure being lower in the erect posture than in the lateral posture may be another triggering mechanism for FI within daily life in patients with CRAI.

Johonson et al. [9] showed that resting pressure was significantly higher in the erect position than in the left-lateral position in the study of healthy adults and attributed this to a higher intrarectal rectal pressure in the erect position than in the left-lateral position. The authors speculated that the change in pressure in the rectum and anal canal might be linked; rising intrarectal pressure challenges the muscular anal canal continence mechanism causing a corresponding rise in resting anal canal pressure. Thekkinkattil et al. [8] reported similar results in a study of 135 patients with FI, ascribing to it to engorgement and bulking of the anal cushion in the erect position compared with the lateral position because the anal cushions are important in contributing to the rise in pressure. Besides patients with FI, those with non-prolapsing hemorrhoids also have a thicker anal cushion and, thus, a higher resting pressure [22]. This study also showed similar results in all 80 FI patients. However, the difference in the median resting pressure measured in the erect position from that in the left-lateral position was only 7–8 cm H_2_O in either the CRAI or non-CRAI patients, which would be clinically insignificant to avoid FI especially in the CRAI patients, because the median squeeze pressure was nearly 30 cm H_2_O lower in the erect position than the lateral position. Anal canal length in the lateral position was longer in healthy subjects compared with female patients with FI in previous studies [23, 24]. Meanwhile, there was no change in the anal canal length with a change in the posture in the healthy adults [9]. In this study, it is unclear why FI patients presented a shorter anal canal length in the erect position compared with the lateral position. However, the difference in the median length was only 3 mm, hence it is uncertain whether this finding might provide additional information on increasing the chance of FI in the erect position.

Studies on the effect of posture on rectal sensation are limited. Halani et al. [25] reported that although there was no significant difference in the rectal volume measured in the left-lateral position compared with the lithotomy position in female FI patients, higher values for the measurements were observed in lithotomy than in the left-lateral position. Another study of urogynecological patients reported similar results [26]. In these studies, testing was performed first in the left-lateral position and then in the lithotomy position. In our study, DDV and MTV were significantly higher in the erect position than in the left-lateral position in the 80 FI patients. The reason is unclear however, but testing was performed in the same sequence as previous studies [25, 26], first in the left-lateral position and then in the erect position in our study, which may have blunted rectal sensation during rectal capacity examination in the erect position. The order of examination should be reversed and re-examined in the future. These results do not seem to have an influence on FI.

This study has several limitations. The major limitation to our manometric technique is the necessity that “pull-throughs” be performed. A manometric device capable of simultaneously measuring radial and longitudinal anorectal pressure profiles would be helpful. The subjects who were continent of feces were not studied as a control group. The involuntary contraction of the internal anal sphincter and the external anal sphincter continence reflex were not measured. The CRAI patients were small in number, and non-CRAI patients had various defecographic findings, including rectocele, which is also regarded as a cause of FI y[27, 28]. Small number of FI patients with IBS were included, possibly because majority of patients with IBS did not consult a surgeon, but a medical physician. Also, the study population came from a single tertiary care center, and our findings may not be generalizable to all patients with CRAI and FI.

In conclusion, voluntary contraction in female FI patients with CRAI was suppressed in the erect position. Further studies that measure the involuntary contraction of the internal or external anal sphincter in these patients are required to explore the whole incontinence machinery.

## Supplementary Information

**Additional file 1**. Eighty female patients with fecal incontinence (FI) who underwent defecography

## Data Availability

All data generated or analysed during this study are included in this published article [and its Additional file 1].
